# Headache and Neuralgia

**Published:** 1889-02

**Authors:** 


### Headache and Neuralgia. By J. Leonard Corning, M. A., M. D. Pages 230. Price, $2.75. New York: E. B. Treat & Co. 1888.

In this volume we have a review from an allopathic standpoint of the pathology and treatment of headache, neuralgia, and morbid sleep. The treatment is chiefly by means of "sedatives," by using electricity, etc.

